# Progress in Otology

**Published:** 1896-07-11

**Authors:** 


### PROGRESS IN OTOLOGY.

(*Continued from page* 228.)

**Glandular Hypertrophy in Aural Disease.**—Behrens[7] insists that the deafness accompanying a common cold is not produced by extension of the catarrh to the middle ear, but rather by indrawing of the drumhead, the consequence of rarefaction of air in the nasal cavities; and he further maintains that in chronic nasal catarrhs, as a rule, the disease is not extended to the ears. On the contrary, he regards the nasopharynx as a safeguard against such extensions. He states that affections of the nasopharynx are the product of the symptoms which diseases of the nose evoke, and not of the disease itself, rarefaction of the atmosphere in the cavity being responsible for most of them. He asks if it is not strange, considering how common occlusion of the nasal passages is, that diseases of the nasopharynx are so rare; and his explanation is that that cavity is but little inclined to pathological change, and gifted with more regenerative power than either the nostrils or Eustachian tubes. Behrens considers that real occlusion of the Eustachian tubes by thickening of the mucous lining, though an occasional occurrence, is a comparatively rare condition, and that agglutination of the walls by mucus is the true explanation of the difficulty we so often meet with in inflating the middle ear. Referring to adenoids, Behrens tells us that there is not a single instrument properly constructed for operation in this region, on account of the irregular shape of the nasopharynx, this irregularity being due to the pharyngeal ends of the Eustachian tubes, which will always remain a stumbling-block to inventors. He therefore depends entirely upon the finger, and in the after-treatment employs for cleansing purposes the douche conducted from a water-bag suspended on the wall with hose and tip which he applies to the least open nostril.

**The Stacke-Zaufel Operation for the Radical Cure of Otorrhœa.**—Luc[8] has published the results of his experiences in a series of fourteen cases in which he employed this method. He adopts the principle of never opening the attic without opening the antrum at the same time, and his method for ascertaining whether the latter participates in the disease is by injecting fluid into the aditus by means of Hartmann's canula after clearing the tympanum, and noting whether it gives rise to the expulsion of a fresh quantity of pus. He is also guided by the presence of a fistula leading from the antrum to the outer surface of the mastoid, or to the posterior wall of the meatus, and by the external evidences of mastoiditis. Luc has discarded Stacke's method of dealing with the membranous meatus, and substituted Zaufel's. As soon as the postero-superior portion of the circumference of the osseous meatus has been exposed by the elevator, he incises the postero-superior half of the membranous meatus at right angles to its length and at the level of the point of entrance into the bone; then by means of a bistoury and dissecting forceps he resects completely the postero-superior part of the osseous meatus. The next important point dealt with by Luc is the question whether the opening is to be maintained temporarily or permanently, or whether immediate closure is to be adopted. In three cases the antrum, which was greatly enlarged by the destructive action of the disease, occupied almost the whole of the mastoid, so that after the opening had been made there was an enormous cavity behind the ear which would never have filled up. This was, therefore, a case for Siebenmann's method for the maintenance of a permanent orifice. In two of the cases the superior and inferior flaps were cut by Kretchmann's modification; in the third one, a single long triangular flap was made. When the mastoid cavity is extremely small and the auditory meatus unusually large, the retro-auricular wound may be closed at once and dressings practised through the meatus. In these cases healing may be brought about in a brief period. On the other hand, from ten to sixteen weeks should be allowed when the extent of the disease of the bone has been very considerable and the consequent opening large, especially if cholesteatomatous masses have been encountered. Nevertheless, the method of epidermisation of the walls of the cavity has diminished the time once considered necessary in cases in which one has had to deal with a cholesteatoma.

**Subcutaneous Phlegmonous Œdema Surrounding the Ear, accompanied by Unilateral Facial Paralysis.**—Gellé[9] has collected a series of cases presenting this combination of symptoms. The œdema of the face precedes the paralysis, and may be situated either on the side of the face, the head, or upper part of the neck. In one case there was dulness of the left ear, a slight sore throat, and on the same side of the face and mastoid region some hot, painful tense swellings. These subsided in about a week, and paralysis of the left facial nerve supervened, followed in about twenty-one days by an attack of gout in the big toe of the left foot, and then the previous symptoms disappeared. An interesting feature in these cases is the localisation of the symptoms principally in the periotic region, and the simulation of mastoiditis occasionally observed, owing to the projection outwards of the auricle. The glands are not as a rule enlarged, and the loss of hearing power is more the effect of swelling of the upper wall of the external meatus than of any lesion of the middle ear. Gellé seems to think these symptoms are attributable to an osteo periostitis of the petrous bone, either of rheumatic or infectious origin. All the forms terminate in resolution. For treatment, ice compresses, quinine in large doses, and sedatives at night have been employed.

**Ear Disease and Nystagmus.**—Urbantschitsch[10] points out the influence of the ear upon the oculo-motor apparatus, and finds that nystagmus is the most frequent affection manifested. More rarely he has noted spasm or paralysis of the ocular muscles or some affection of the internal muscular system of the globe. The nystagmus (usually the rotatory form) may result from disease of any portion of the auditory apparatus, including the external meatus and acoustic nerve. Oscillation of the eyeball has been observed to accompany slight tympanic inflammation, whilst a more violent degree has set up rotation. Nystagmus has also been evoked by slight causes, *e.g.*, syringing the ear, particularly with cold water, so that pressure is not the only agent, and thermic influences may have a similar effect. These phenomena may be reflex in origin, or induced directly by morbid conditions of the occipital lobes or by sinus thrombosis. A much rarer consequence of aural disease is strabismus, of which Urbantschitsch records two cases. A boy aged six suffered from convergent squint, which was directly attributable to the otitis media, and varied in degree with the intensity of the otitis. When the ear affection was cured, the strabismus was much improved, but not quite obviated. Another singular case was that of a woman in whom divergent strabismus resulted from an aural polypus. Removal of the growth with a cold snare caused sudden increase of the squint, which persisted after the ear had got quite well.

Similar instances have been recorded by Lucœ and Moos. Dilatation of the pupil has been observed to accompany the removal of polypi, and the condensation and rarefaction of the air in the external auditory meatus of patients having perforations of the tympanic membrane.

**The Localisation of Inflammations of the Temporal Bone**—Lubet-Barbon,[11] who has collected observations upon this subject by Bezold, Kirchner, and others, points out that these inflammations (osteites) are occasionally limited to the developmental centres of the bone—petro-mastoid, squamous, and tympanic. The lesions are in these cases in fact confined anatomically within the boundaries constituted by the obliterated fœtal sutures. This is, however, quite exceptional, and more commonly, especially in chronic cases, the affection is situated at the point of coalescence of the three divisions of the bone at the level of the antrum, and one ought to work from this point towards the centres in order to follow up the track of the disease.

**Supposititious Foreign Bodies in the External Auditory Meatus.**—The following almost unique case occurred in Milligan's[12] practice. Whilst a workman was engaged in running molten metal into sand troughs, one of his fellows in passing upset the ladle, and a small quantity of the metal splashed up and entered his right auditory meatus. Intense pain was felt, and he was at once sent to the nearest hospital, where attempts were made to extract the metal supposed to be in the ear *without any previous examination of the part.* Probes and forceps were used, and ultimately a small white bone was extracted, causing such severe pain and collapse that the patient had to be put to bed and stimulants administered. The next day he was sent to the ear institution, and the following was the condition found: Upon the postero-inferior meatal wall was an eschar of considerable size, and bare bone was felt with the probe. At the bottom of the meatus was some blood and semi-purulent fluid. The greater part of the membrane was gone, and the manubrium was broken away from the short process. The incus could not be seen, and the tympanic mucous lining was congested and covered with scattered hæmorrhages. The hearing power on the affected side was *nil.* The parts healed rapidly under a sedative lotion, but only very slight improvement took place in the hearing. Probably in this case the only injury done by the molten metal was excoriation and exposure of the bone upon the postero-inferior wall, and this surface was mistaken for a foreign body. Milligan has frequently been consulted in cases where attempts at removing a foreign body have resulted in pushing the latter through the membrana tympani. The three following cardinal maxims given in his paper for guidance in removing such bodies should be laid to heart by every general practitioner. These are: "(1) Be sure that a foreign body really exists in the meatus; (2) if present, make a careful attempt to remove it with the syringe; (3) if instruments have to be resorted to, be careful that the work is done under proper illumination, and with the utmost gentleness."

**The Aural Complications of Leukæmia.**—Orne Green[13] has recently examined three cases of well-marked leukæmia in which there were aural symptoms present. The first was that of a woman aged forty, in whom there was great enlargement of the spleen, much headache, and occasional vomiting; there was a great increase of the large mono-nucleated lymphocytes. Two months later she had a severe attack of vomiting with vertigo followed immediately by deafness in the right ear, and a continuous roaring with more or less pain in the head. Examination of the ears showed slight retraction and opacity of both drum membranes, and great diminution of both air and bone conduction on the right side. These symptoms as well as her general condition became gradually worse. The next case, a woman aged twenty-three, with enlarged spleen and having a proportion of red corpuscles to white, as 1·2, entered hospital in January, and in February following had a sudden attack of dizziness with rotation of objects from left to right, and noises in the head. Tuning-fork tests gave Weber wholly in the left ear, bone conduction diminished in the right, and hearing for voice and watch diminished on that side, but normal on the left. The hearing in this patient improved. The third case was that of a male patient with an enormously large spleen, almost totally deaf, and complaining of constant buzzing in the ears; his general condition improved very much, but the aural symptoms remained the same. The morbid conditions affecting the ears in leukæmia are exudative and hæmorrhagic processes in the middle or internal ears, and slight hæmorrhages in the meatus and drum membrane. The labyrinth is the most frequently affected. According to Haug the otitis begins suddenly, sometimes in one side only, oftener in both together. Vertigo is always present, with staggering gait and other disturbances of co-ordination, also metallic ringing and occipital headache. Great and often complete deafness follows the vertigo almost immediately, it may improve but is liable to recur at intervals. There is no treatment of any avail for the aural symptoms any more than for the general disease, and we can only hope that absorption of the effusion may take place, which is possible if sufficiently small in amount.

**The Removal of Ceruminous Accumulations.**—Laurens[14] insists on the absolute avoidance of all instruments in attempting dislodgment of the mass. The syringe employed should be thoroughly sterilised, its extremity fine and perfectly cylindrical, but all the better for having a soft rubber tube, one centimetre in length, attached to one end, in order to prevent all risks of injury to the passage. He deprecates violent syringing if the plug is obstinate in coming away, and thinks this practice may injure the labyrinth, or by causing sudden detachment of the mass from the external surface of the drum membrane may set up hæmorrhage. He, therefore, recommends the usual solvent instillation of glycerine and bicarbonate of soda to be dropped into the ear three times a day, followed by a tampon of cotton. The increase of deafness, buzzing, and vertigo which are apt to supervene as the cerumen swells up in the meatus may be neglected, as they will disappear when the mass is dissolved. These are excellent rules for the guidance of those who are not experienced in aural manipulations.

[7] Int. Clin., iv., 3. [8] Proc. French Soc. of Lar. and Otol., reported in Jnal. of Lar., Feb., 1896. [9] Ibid. [10] App. B. M. J., Feb. 15, 1896. [11] Jnal. of Lar., Feb., 1896 [12] Med. Chron. Mar., '96. [13] Int. Clin., vol. iv., ser. 4. [14] N. Y. Med. Jnal., Mar. 7, 1896.

